# Supplementary material for: Are carbon emissions trading and green financial instruments synergistic? -Comprehensive quantitative research based on content analysis
Source: PLoS One. 2024 Mar 7;19(3):e0298601. doi: 10.1371/journal.pone.0298601 (PMC10919601; doi:10.1371/journal.pone.0298601)
Supplement: S1 Data — (ZIP) [file pone.0298601.s001.zip › Data/地级市Z_F工作报告.zip!/╔┬╬≈/╔╠┬σ╩╨/└┤╫╘╣1⁄2╓┌║┼í╛┬φ┐╦╩2╛▌═°í┐_2018.docx]

各位代表：

现在，我代表市人民政府向大会报告工作，请予审议，并请市政协委员和列席人员提出意见。

一、2017年工作回顾

2017年是商洛发展进程中砥砺奋进的一年。在省委、省政府和市委坚强领导下，我们紧紧依靠全市人民，牢牢把握稳中求进工作总基调，全面落实追赶超越和“五个扎实”要求，大力实施“五新”战略，全力推进“五大攻坚战”，较好完成了年度各项任务，全市经济社会发展焕发新活力、迈上新台阶。

追赶超越步履坚实。生产总值增长9.5%，规模以上工业增加值增长14.5%，增速均居全省第三；城乡居民收入分别增长8.6%和9.3%，全社会固定资产投资增长23.6%，增速均居全省第二；非公经济占比55.2%，位居全省第三；地方财政收入同口径增长9.1%。160个市级重点项目完成投资580亿元，占年任务122.6%，山柞高速、镇安抽水蓄能电站、万达广场城市综合体等重大项目加快建设，比亚迪电池材料、必康中药中间体提取等48个项目建成投产。特别是，事关商洛追赶超越发展的“十件大事”取得重要进展:盘龙药业在A股中小板上市，创深度贫困地区企业通过IPO绿色通道上市等“四个第一”，18家企业在陕西股权交易中心挂牌;西十、西康两条高铁和丹宁、榆商、洛卢三条高速前期工作进展顺利；商洛国家级高新区创建列入科技部培育计划；金凤新区一期项目形象展示区建设工程全面开工；天竺山等5A级景区创建加快推进；牛背梁、漫川关获评省级旅游度假区；丹江百里生态长廊概念性规划编制完成；主城区集中供热完成地勘招标；“气化商洛”有效推进，商州至洛南段管线敷设基本完成，商州至商南、山阳段开工建设。

五大攻坚战成绩斐然。大力实施精准扶贫脱贫，全面落实“八个一批”，培育新型农业经营主体6186个，带动6.2万户群众发展特色产业，5.1万名搬迁群众住房有保障，“三带一创”产业精准扶贫和易地扶贫搬迁“三精”工作模式全省推广，创业就业、危房改造、教育、健康、生态和兜底扶贫精准有力，宁商扶贫协作和国资、高教、卫计三大帮扶体系添力助力，农村基础设施条件不断改善，6.69万人稳定脱贫，53个村退出贫困行列。力促现代工业转型升级，出台中国制造2025商洛实施方案，实施新一轮企业技术改造，50个市级重点工业项目完成投资120.8亿元，新培育产值过亿元企业26家、规模以上企业31家，新发展省级“三星梯队”企业42家、企业技术中心2个、“双创”基地3个，绿色食品、现代医药产值增长30.1%和30.9%，战略性新兴产业增加值增长15.6%。推动特色农业提质增效，“商洛核桃”入选中国特色农产品优势区，“洛南核桃”“洛南豆腐”“云盖寺挂面”成为国家地理标志保护产品，“商洛香菇”获国家农产品地理标志登记产品，创建全国农村创业创新园区14个，新认定省级农业产业化龙头企业6家，丹凤棣花镇、山阳枫树村列为全国“一村一品”示范镇村，商州江山村获评中国美丽休闲乡村。全面启动全域旅游示范市创建，率先在全省组建市县区旅发委，成功举办秦岭生态旅游节十大品牌活动，金台山晋升4A级景区，终南山寨等“旅游+”项目建成运营，营盘镇入选全国首批运动休闲特色小镇，全年接待游客人数和旅游综合收入分别增长28%、30.4%。加快新型城镇化发展步伐，“五城联创”扎实推进，“四大门户区”等项目顺利实施，江滨银杏公园建成，环城南路隧道贯通，迎宾大道、北新街、商洛路完成路面改造，中心城市绿化美化亮化取得新成效，13个棚改项目全部开工。县城建设全面提速，丹凤荣膺全省县城建设先进县。特色小镇建设走在全省前列，漫川关镇、云盖寺镇入选第二批全国特色小镇。全市户籍人口城镇化率达到53.7%。

生态商洛优势凸显。国家循环经济示范城市加快创建，淘汰落后产能等30项指标超额完成。生态建养不断加强，植树造林36.3万亩，治理水土流失面积703平方公里，“河长制”“田长制”全面落实。张坪、磨沟、云镇等水库建设进展顺利，20.71万人饮水安全得到保障。中省环保督察巡查问题有效整改，丹江等流域污染防治四年行动计划扎实推进，主要河流出境断面水质稳定达标。强力开展铁腕治污降霾，中心城区空气优良天数331天，稳居全省第一。农村垃圾就地化、减量化、资源化处理经验全省推广，山阳前店子村获评全国改善农村人居环境示范村。

改革开放活力增强。供给侧结构性改革扎实推进，取缔“地条钢”产能9.6万吨，商品房去库存7941套，争取企业直供电14.69亿度。“放管服”改革持续深化，市场秩序整治有力有效，取消、下放市级行政审批事项27项，新增市场主体14722户，民间投资增长43.8%。农村土地确权登记全面完成，“三变”改革试点顺利实施，11.4万农民变“股东”。创新型商洛建设成效显著，高新技术企业达到17家，技术合同交易额1.09亿元。丝博会、陕粤港澳合作周和县区客商大会等活动成果丰硕，维维集团核桃产业深加工等重大招商项目成功落地，全市招商到位资金651.28亿元，增长26.5%。服务业加快发展，供销综合改革稳步实施，新增限上服务业企业58家，社会消费品零售总额增长10.8%，进出口总额21.69亿元，实际利用外资1544万美元。

民生福祉持续改善。“两个80%”民生政策全面落实。新增城镇就业1.9万人，农村劳动力转移就业47.4万人，省级创业型城市通过验收。教育优质均衡、素质教育和学校安全标准化全面推进，高考二本以上上线率连续10年稳居全省前列，整市通过“双高双普”评估验收。医药卫生体制改革不断深化，县域内就诊率90.14%。政府购买医疗救助服务稳步开展，新建社区服务中心49个、农村互助幸福院150个。花鼓戏曲电影《带灯》成功首映，《紫荆树下》《孝义川》荣获省“文华优秀剧目奖”，环秦岭公路自行车赛、四国男篮挑战赛等十大精品赛事成功举办。“七五”普法深入开展，食品药品安全监管全面加强，安全生产形势总体平稳，平安建设和信访“三无”县区创建扎实有效，群众安全感不断增强。

过去一年，我们竭力办好中心城区民生“十件实事”，取得了较好成效：一是新改扩建中小学、幼儿园15所，去年秋季投用6所，新增学位8400个；二是建成市政务服务中心，347项行政审批服务事项实现“一厅式办理”，县区政务服务中心投入运行；三是规划新建停车场4个，新增泊车位5096个；四是推进出租车行业和公交车运营改革，城东客运站全面竣工，新能源电动公交车和“一卡通”正式投用；五是加快东新路、东环路等农贸市场建设和团结路改造，黄沙桥市场整体搬迁，江南小区和东环路小吃城基本建成；六是建成垃圾转运、收集站点71座，购置垃圾清扫清运车205辆，新改建二类公厕78座，改造旱厕84座；七是丹江绿道、江滨银杏公园健身长廊和6个小广场建成投用，市体育馆对外开放；八是完成城周高标准造林2.1万亩；九是积极推进文化惠民工程，开展群众性惠民演出52场次；十是完成黄沙河、柳家沟河道治理，马莲峪河道治理基本完成。

过去一年，我们着力加强政府自身建设，深入推进“两学一做”学习教育常态化制度化，全面落实“三项机制”，严格执行八项规定，持续纠正“四风”问题,不断加强党风廉政建设,“三公经费”同比下降7.58%。深化法治政府建设，全面完成政府规章和规范性文件清理。自觉接受人大依法监督、政协民主监督，代表议案建议、委员提案办复率均达100%。重视审计监督、舆论监督和社会监督，积极回应各方关切，持续加强效能建设，政府执行力、公信力不断提升。同时，国防动员、民族宗教、外事侨务、防震减灾、妇女儿童、气象、水文、档案、统计、人防、方志、老龄、无线电等工作取得了新成绩，慈善、红十字会、残疾人和关心下一代等事业都有了新发展。

各位代表！过去一年，面对复杂多变的宏观形势，我们取得这样的成绩实属不易，这是省委、省政府和市委正确领导的结果，是市人大常委会、市政协监督支持的结果，是各民主党派、工商联和无党派人士广泛参与的结果，是全市干部群众共同努力的结果。特别是在脱贫攻坚主战场上，广大党员干部舍小家、为大家，肩负使命重任，心系贫困百姓，付出了大量心血，甚至献出了宝贵生命。在此，我谨代表市人民政府，向全市广大干部群众、驻商部队指战员、武警官兵和公安干警致以崇高敬意！向关心支持政府工作的各位代表、各位委员和社会各界人士表示衷心感谢！

二、2018年思路目标

2018年是贯彻党的十九大精神开局之年，是改革开放40周年，是决胜全面小康、实施“十三五”规划承上启下的关键一年。中国特色社会主义进入新时代，对政府工作提出了新要求。我们必须按照抓首要、大学习、促发展的要求，深入学习、深刻领会、全面贯彻习近平新时代中国特色社会主义思想和党的十九大精神，坚持问题导向，强化责任意识，弥补发展短板，破解发展难题。

知不足而奋进，弥差距惟自强。当前，商洛发展还面临不少困难和挑战，发展不平衡不充分的问题依然存在，特别是不充分问题尤为突出，主要是：实体经济薄弱，市场主体偏少，规模企业不多；产业链条较短，低端产业较多，高附加值产品较少；高新人才匮乏，科技成果转化率不高，创新发展不足；城乡基础设施欠账较大，区域发展不平衡，产业结构不合理，社会治理水平不高等等。

同时，我们要充分认识到，商洛目前正处于大有可为的历史机遇期，蕴含着诸多发展利好：从国家战略层面看，支持川陕革命老区振兴、实施乡村振兴战略、加强东西部扶贫协作等一系列重大举措，尤其是国务院批复关中平原城市群发展规划，为我们加快融入大西安都市圈，建设“一带一路”重要节点城市，提高人口和经济聚集水平，构建现代化经济体系，推动高质量发展提供了难得机遇；从全省发展布局看，省委、省政府大力发展枢纽经济、门户经济、流动经济，推行“一市一策”、强化陕南循环经济园区建设、创建国家级生态文明改革试验区等重大部署，为我们加速推进生态、区位、交通等优势向经济优势、发展优势转化提供了难得机遇。为此，我们必须坚定信心、下定决心，顺势而为、乘势而上，奋力开启新时代商洛追赶超越新征程！

今年政府工作的总体思路是：全面贯彻党的十九大精神，以习近平新时代中国特色社会主义思想为指导，以推动高质量发展为目标，以脱贫攻坚统揽经济社会发展全局，坚持稳中求进工作总基调，坚持新发展理念，对标追赶超越和“五个扎实”，认真落实“五新”战略任务，持续打好“五大攻坚战”，扎实做好改革发展各项工作，推动质量变革、效率变革、动力变革，促进经济社会持续健康发展。

预期目标是：生产总值增长9%左右，规模以上工业增加值增长12%左右，全社会固定资产投资增长12%左右，社会消费品零售总额增长10%左右，地方财政收入同口径增长6%左右，城乡居民收入分别增长8.5%和9.3%左右，城镇登记失业率、居民消费价格指数分别控制在3.9%和3%以内，生态环保和节能减排指标控制在省定范围内。

各位代表，目标需要实干来完成。我们必须以永不懈怠的精神、舍我其谁的担当、一往无前的勇气，凝聚“三个商洛”建设的磅礴力量，干成一批经得起时代和人民检验的实事。

三、2018年重点任务

（一）坚持精准施策，不断提高扶贫脱贫质量。必须更加清醒认识脱贫攻坚的艰巨性，按照中省部署要求，尽锐出战，精准施策，确保全年完成20万人、358个贫困村脱贫摘帽任务。

突出抓好产业扶贫。继续深化“三带一创”，完善产业扶贫利益联结机制，扶持壮大新型农业经营主体，每县区培育2-3个全链条特色产业，推进旅游、光伏、科技、电商等多种形式扶贫，确保50%以上贫困人口通过发展产业稳定增收。创新金融扶贫，积极开展“卢氏模式”试点，建立市、县产业脱贫发展基金，加大涉农资金整合力度，把60%以上扶贫资金用于产业脱贫。

改善设施助推脱贫。争取中省深度贫困地区政策和项目资金支持，实施通村通组水泥路、安全饮水巩固提升和自然村通动力电等脱贫攻坚基础设施“三提升”工程。加大易地扶贫搬迁和农村危房改造力度，推进宜居、宜业“双示范”社区创建活动，年底前易地扶贫搬迁安置房基本达到入住条件，完成建档立卡贫困户危房改造8063户。推进贫困村标准化卫生室、义务教育学校、残疾人康复和托养服务等设施建设，提高贫困村公共服务水平。

大力推进社会扶贫。抓好宁商扶贫协作和经济合作框架协议落实，促进土地增减挂钩指标流转，加快商州笃诚医药产业园等12个重点产业协作项目落地实施，选取有一定基础的园区、镇村发展“飞地经济”和村级集体经济项目。加强与国资、高教、卫计三大帮扶体系对接联络，深化“万企帮万村”精准扶贫行动，用好中国社会扶贫网平台，引导社会各方力量参与脱贫攻坚。

完善脱贫政策保障。开展“脱贫攻坚作风建设年”活动，建立健全稳定脱贫长效机制，保证现行标准下的脱贫质量。认真兑现教育扶贫、健康扶贫、生态扶贫、资产收益扶贫等政策，抓好社会保障兜底扶贫，实现农村特殊人群“应保尽保”“应养尽养”。采取公益性岗位安置、组织转移就业等措施，帮助贫困家庭就业增收。注重扶贫与扶志、扶智相结合，引导树立勤劳致富、脱贫光荣的导向，让贫困群众敢想敢干、愿想愿干、能干会干，加快补齐“精神短板”，激发贫困人口内生动力。

（二）扩大有效投资，促进经济持续稳定增长。发挥投资关键性作用，牢牢抓住项目建设“牛鼻子”，精准开展招商引资，筑牢县域经济发展基石。

狠抓重大项目建设。持续推进“十件大事”，抓好180个市级重点项目建设，加快必康国家中药材战略储备库暨大数据交易平台、万达广场城市综合体、秦煌年产10万辆纯电动汽车等78个续建项目，山柞高速年内建成通车。推进盘龙药业技改等102个新建项目，力促西十、西康高铁早日开工。抓实150个重大项目前期工作，力争丹宁、榆商、洛卢高速等50个以上项目具备建设条件，做好商郧高速前期谋划工作。紧盯国家政策导向，全年策划储备重大项目不少于400个。

提高招商引资成效。精心筹备参加2018丝博会和陕粤港澳合作周，策划举办第六届陕南循环经济合作交流等主题招商活动，支持县区办好客商大会。瞄准国际国内500强和京津冀、长三角、珠三角等地区，开展招商引资、引才引智，鼓励“商洛能人”返乡创业。健全完善招商项目落实机制，提高项目履约率和资金到位率，全年招商引资到位资金660亿元。

持续壮大县域经济。统筹县区产业发展，推进形成资源协同、优势互补、错位发展的区域产业新格局。支持商州和柞水发展食用菌产业、商南建设茶产业转型升级示范县、山阳打造国家现代中药材产业基地，鼓励洛南和丹凤争创核桃产业转型示范县、镇安发展板栗产业。加快县域产业园区建设，创建3个以上省级示范县域工业集中区。大力发展民营经济，当年新增各类市场主体8000户，非公经济占比55.5%。加快推进洛南撤县设区。鼓励县区争先进位，筑牢经济发展“底部支撑”。

着力改善营商环境。实施“营商环境提升年”行动，开展降低企业运行成本、简化施工许可证办理程序、县域营商环境监测评价等十大行动。深化“放管服”改革，实现“双随机、一公开”监管全覆盖，全面清理涉企收费项目，规范市场秩序。推进市县区政务服务大厅和网络平台规范化建设，建成市“12345”便民热线综合服务平台，开展“减证便民”专项行动，建立全市证明事项和盖章环节清单，打通服务群众“最后一公里”。

（三）实施乡村振兴战略，推进农业农村现代化。坚持农业农村优先发展，按照产业兴旺、生态宜居、乡风文明、治理有效、生活富裕的要求，推动农业全面升级、农村全面进步、农民全面发展。

发展特色现代农业。开展粮食生产功能区划定，粮食总产稳定在55万吨左右。扩大特色产业规模，新增核桃示范园7万亩、中药材基地32万亩、生态茶园2万亩、代料食用菌1.8亿袋、苗木花卉1万亩，板栗嫁接改造8.5万亩。发展新型农业经营主体，新增市级以上现代农业园区、龙头企业、示范合作社、家庭农场各10个，培育新型职业农民1800人。用好“国家农产品质量安全市”金字招牌，加快国家农业科技园区和特色农产品优势区创建，推进“三品一标”认证和标准化生产，扩大商洛优势农产品牌影响力。支持发展乡村旅游、文化创意、农村电商等新产业、新业态，促进农村一二三产业融合发展，提高农业效益和农民收入。

建设美丽宜居乡村。编制乡村规划，实现行政村规划编制全覆盖。改善农业农村基础条件，新修改造基本农田4.5万亩，开工建设水库4座，新增高效节水灌溉面积3.2万亩，巩固提升20万人饮水安全。建设“四好农村路”，整治通村公路“油返砂”路段918公里，实施村级道路硬化501公里。新增无线网络覆盖403个村806个点、农村有线电视用户5.63万户。整治农村人居环境和村容村貌，持续实施化肥农药使用零增长行动，创建美丽宜居示范村50个。

深化农村各项改革。落实第二轮土地承包到期后再延长30年政策，加快土地“三权分置”改革，推进土地有序流转，发展适度规模经营。抓好集体产权制度改革，健全县级产权交易平台，30%的村（社区）完成清产核资，新增“三变”改革示范村200个。深化集体林权制度改革，完成国有林场改革任务。完善农村金融保险，推进政策性农业保险增品扩面，落实税费、电价等优惠政策，鼓励引导工商资本参与乡村振兴。健全自治、法治、德治相结合的乡村治理体系，持续推进农村移风易俗。

（四）加快转型升级，推动工业发展质量变革。坚持依托资源优势，培育壮大支柱产业，加快发展实体经济，着力构建现代工业体系。

认真落实中国制造2025商洛实施方案。紧扣“763”行动方案和分产业行动计划，发挥战略规划领导小组和8个产业推进组作用，滚动实施100个重点支撑项目，培育打造优势特色产业新体系。抓好海恩动力电池正极材料、国漆科技产业园、光电产业园等50个重点工业项目建设，实施30个企业技改项目，推进中药配方颗粒、非金属产业延链等前期项目，策划包装10个投资超10亿元的工业项目，延长一批重点产业链条。

壮大工业实体经济。培育壮大骨干龙头企业，大力扶持本土企业，每县区发展产值过亿元企业2户以上。实施中小企业成长梯队计划，落实新纳规企业奖励政策，新增规模以上企业25家。全面落实“工业17条”，深入开展企业包扶活动，支持骨干企业满负荷生产，促进新建项目释放产能，帮助新纳规企业扩大规模。

加快园区提档升级。积极创建商洛国家级高新区，推进洛河新型工业园区建设，支持山阳省级高新区创建。高质量编制工业园区产业发展专项规划，明确园区主导产业、辅助产业和产业布局，建成一批产业对口协作基地和小微企业孵化园。健全投融资平台，推动金融机构、金融产品、金融服务进园区。加强园区管理，完善基础设施，提高生产性服务水平，新建标准化厂房20万平方米，新增入园企业40家。

（五）狠抓全域旅游，提升服务业发展水平。抓住国家支持深度贫困地区旅游扶贫行动政策机遇，实施全域旅游基础改善、服务提升、品牌推广等工程，带动服务业健康发展。

着力提升景区品质。扩大金丝峡5A级景区品牌效益，推进牛背梁、天竺山、金台山5A级和九天山、音乐小镇、天蓬山寨、童话磨石沟等4A级景区创建，加快牛背梁、漫川关国家旅游度假区创建。抓好洛南锦绣大地、丹凤飞行小镇、商南富水田园小镇、山阳丰阳印象等项目开发，商州江山景区、丹凤上运石地质公园等投入运营。建成市县区游客中心、散客集散地和大数据中心。加大旅游市场监管，优化旅游服务环境，提升游客满意度。

持续实施“旅游+”战略。加快商於古道等文化旅游项目建设，大力发展红色旅游，完善旅游要素，丰富旅游产品，培育特色民宿、体育健身、康体养生等业态，推进旅游业与农业、健康、文化、体育等产业深度融合。策划举办2018中国秦岭生态旅游节等十大品牌活动，与周边城市建立旅游营销联盟，开发跨省市精品线路，规范发展旅行社，提升“秦岭最美是商洛”品牌影响力，全年接待游客人数超过5000万人次，旅游综合收入突破300亿元。

加快服务业发展。提升传统服务业品质，发展现代物流、电子商务、健康养老、家政服务、物业管理等产业，培育融资租赁、众创众筹等新业态，新增限上服务业企业85家，服务业增加值增长8%左右。抓好市电商仓储物流配送中心、西北核桃交易中心、镇安高山农产供应链等项目建设。推进县级供销合作社综合改革，加快发展供销合作经济。

（六）突出扩容提质，扎实推进新型城镇化。坚持高起点规划、高标准建设、高效益经营、高水平管理，加快推进新型城镇化发展步伐，全市户籍人口城镇化率提高1.2个百分点。

提升城乡规划管理水平。完成中心城市和各县县城总规修编，启动城市设计编制，加快各县县城控规编制。严格规划审批监管，深化城管执法体制改革，加大城市违法建设和环境综合整治力度，建设市县智慧城管平台。整顿规范房地产市场秩序，建立多主体供给、多渠道保障、租购并举的住房制度。贯彻落实《商洛市住宅物业管理条例》，规范提高城市物业管理服务水平。

推进中心城市提质建设。注重城市功能完善，推动“一体两翼”中心城市内涵式发展。深入推进“五城联创”，实施道路、公园广场和绿化美化亮化提升工程，国家卫生城市通过省级评估并报国家验收，省级生态园林城市通过验收并申报创建国家园林城市。加快金凤新区、南秦新区、商洛高新区建设，推进麻街至构峪桥城市干线、高新区紫荆遗址公园等项目建设，完成工农路、文卫路北段提升改造工程，中心城区集中供热项目主体工程基本建成，市植物园、南秦湿地公园一期等建成投用。开展“棚改提效年”活动，加快续建和新上棚改项目建设，力争碧桂园智能生态高端住宅和宏帆集团大型城市综合体项目落地建设。

加快县城和特色镇建设。以市政道路、管网改造、污水垃圾处理、绿地广场等项目建设为重点，进一步完善县城基础设施，拉大城市框架，鼓励建设城市综合体和特色街区，提升人口聚集力和产业承载力。加快商州至商南、山阳天然气长输管线建设，推进镇安、柞水县城天然气利用工程。启动第二批秦岭特色小镇建设，全年完成重点镇建设投资16.02亿元。

（七）加大环保力度，建设更加优美生态环境。坚持绿色循环发展不动摇，固守生态红线，扩大生态优势，让绿水青山永续成为造福商洛百姓、惠及子孙万代的金山银山。

推进绿色循环发展。完成国家循环经济示范城市创建验收。大力发展清洁能源，光伏发电装机累计达到500兆瓦，加快商南莲花台、山阳月亮湾、镇安旬河等水电站建设，全年新增水电装机2000千瓦，开工建设商州闫村风电项目，推进镇安北阳山等风电项目前期工作。抓好“两山”理论试验区、“中国生态气候康养（宜居）地”创建和国家气候适应型城市建设试点。

实施生态保护工程。落实重点生态功能区产业准入负面清单，划定生态保护红线，完成秦岭生态环境保护规划编制和全国第二次重点污染源普查，开展全市第三次土地调查。推进天然林保护和退耕还林工程，完成造林32万亩，市区城周绿化5000亩、补植提高2万亩。抓好丹江百里生态长廊等项目建设，治理水土流失面积600平方公里，每县区至少建成1个山水林田湖综合示范项目。落实最严格的水源地保护措施，保障城乡供水安全。

加大环境治理力度。实施丹江等流域污染防治四年行动计划，推进沿江沿河镇村和移民搬迁点垃圾污水处理设施建设，开展中心城区餐厨垃圾无害化处理试点。整治建筑施工和道路扬尘，推进煤炭高效清洁利用，实施清洁替代和电能替代，推动电化商洛，呵护好“商洛蓝”。做好土壤污染防治与修复，强化重金属污染治理。抓好中省环保督察反馈问题整改，着力解决环境突出问题。

完善生态环境监管体制。严格矿产资源开发准入管理，推进绿色矿山建设，鼓励尾矿资源综合利用。加强森林资源保护，开展秦岭生态环境保护专项整治，严格落实生态环境损害赔偿制度和责任追究制度。加强领导干部自然资源资产离任审计，完成环保机构执法垂直管理改革。强化环境监管网格化管理，坚决制止和惩处破坏生态环境行为，守护好商洛的青山绿水。

（八）深化改革开放，不断增强发展的动力活力。推进各项改革任务落实，一以贯之增强忧患意识、防范风险挑战，谱写创新发展、开放发展新篇章。

深化供给侧结构性改革。推进“三去一降一补”，扩大有效高端供给，延长补强绿色食品、新材料、生物医药等产业链。巩固商品房去库存成果，抓好减税降费政策落实，争取直供电15亿度，着力降低企业成本。培育壮大骨干财源，强化税收征管，不断提升收入质量。落实民营促进、金融创新等补短板十大举措，增强发展的协调性、均衡性和可持续性。

推进重点领域改革。深化投融资体制改革，规范推进PPP项目实施，用好“一行三会”支持深度贫困地区脱贫攻坚等政策，拓宽直接融资渠道，支持森弗、丰源钒业、天之润、丰联工贸等企业在多层次资本市场挂牌上市。加快设立城市建设和产业发展基金，县区设立贷款担保基金和风险补偿基金，实现政府性融资担保机构业务县域全面覆盖。深化国资国企改革，完成国企“三供一业”分离移交工作。推进科技、文化、医药卫生和安全生产等领域改革。

防范化解重大风险。全面实施绩效管理，提高财政资金使用效益。强化预算管理，严格政府债务规模限额管理，坚决制止违法违规融资担保行为，严禁以政府投资基金、政府和社会资本合作、政府购买服务等名义变相举债，严控地方政府债务风险。成立市县地方金融监管机构，严厉防范和打击非法集资、金融诈骗等违法犯罪行为，坚决守住不发生系统性金融风险底线。

推动科技创新和对外开放。深入实施质量强市战略，开展质量提升行动，强化质量考核评价。巩固创新型商洛建设成果，推进科技资源统筹、知识产权转移等平台建设，支持企业与高校、科研院所联合开展研发和成果转移转化，推进技术创新、质量创优、品牌创建，培育高新技术企业、瞪羚企业，实施科技成果转化项目10个，创省级以上名牌产品2个。加强人才队伍建设，激发和保护企业家精神，弘扬劳模精神和工匠精神。推动商洛陆路口岸建设，支持比亚迪、君威等企业扩大出口，建设特色产品海外交易市场和展示中心，全年实现进出口总额24亿元，实际利用外资2000万美元。

（九）着力改善民生，持续增进人民群众福祉。坚持以人民为中心的价值追求和执政为民的责任担当，尽力而为、量力而行，多为群众办实事、解难题、添福祉，让全市人民获得感、幸福感更加充实、更有保障、更可持续。

大力发展教育文化事业。把教育放在优先位置，统筹城乡义务教育一体化发展，加快城区中小学校建设，实施义务教育全面改薄等重点项目，推行学校安全标准化管理。落实立德树人根本任务，深化教育综合改革，加快普及高中阶段教育，支持民办教育发展，规范社会力量办学。支持商洛学院与洛南等县区开展校地合作，创建一流应用型本科院校。加快商洛职院一流专业培育和优质校建设。推动文化事业发展，办好首届商洛艺术节，加强文物保护利用和文化遗产保护传承，加快市博物馆选址建设，完成58个村“百县万村”广播设备配送，建成275个基层综合性文化服务中心。

不断提高社会保障水平。健全职工工资正常增长机制，完善最低工资和工资支付保障制度。支持返乡农民工、高校毕业生、退伍军人等群体创业就业，新增城镇就业1.55万人，农村劳动力转移就业40万人。扩大社会保险覆盖面，提高城乡居民基本医疗保险财政补助和个人缴费标准。加快社会养老服务体系建设，完成市中心老年公寓一期工程，新建改建2个县级敬老院和3个区域敬老院，建成城市社区日间照料中心6个、农村互助幸福院100个。实施重特大疾病医疗救助，关爱保护“三留守”人员，大力推进殡葬改革。

深入实施健康商洛战略。实施医疗服务行动计划，加强基层医疗服务体系建设，推进流动人口卫生计生基本公共服务均等化。深入开展爱国卫生运动，全面推进“厕所革命”。推动中医药振兴发展，打造商药品牌，促进医疗与养老、旅游等行业深度融合。启动市中心医院新建项目，完成市残疾人康复中心主体工程，市精神卫生中心一期建成投用。精心办好2018中国环秦岭公路自行车赛、陕西·商洛马拉松赛等十大精品赛事，每县区实施2个全民健身示范工程，启动建设第十四届全运会在商比赛场馆，积极备战第十六届省运会。

打造社会治理新格局。纵深推进“七五”普法，提高依法治理水平。高度重视安全生产，持续强化食品药品安全监管，坚决遏制重特大事故发生。提升信访工作质量，及时排查化解社会矛盾和风险隐患。完善立体化信息化社会治安防控体系，开展新一轮扫黑除恶专项斗争，继续打击多发性侵财犯罪和电信网络诈骗犯罪，巩固无传销市创建成果。支持国防和军队改革建设，积极开展国防教育、国防动员、人民防空等工作，推动军民融合发展，更加细致做好复转军人安置和优抚工作，不断巩固和发展军政军民团结的大好局面。

各位代表，新时代赋予新使命，新征程呼唤新作为。全市政府系统必须坚持党的领导，树牢“四个意识”，加快职能转变，提高行政效能，努力建设人民满意的服务型政府。

我们必须加强学习，增强本领。梦想从学习开始，事业从实践起步。各级政府要大兴学习之风，认真抓好党的十九大精神学习宣传贯彻工作，扎实开展“不忘初心、牢记使命”主题教育，全面增强“八个本领”，切实加强调查研究，注重实践锻炼，以更高的素质和能力适应新形势、落实新理念、推动新发展。

我们必须依法行政，从严履职。政府的一切权力来自人民、源自法授。各级政府要健全学法用法长效机制，严格执行重大行政决策法定程序，运用法治思维、法治方式、法治手段行使权力、履行职责，自觉接受人大依法监督和政协民主监督，主动接受社会和舆论监督，让政府工作在法治轨道上稳步前行。

我们必须心系百姓，实干苦干。大道至简，实干为要。各级政府要时刻把群众的事情放在心上，把发展的重任扛在肩上，坚持干字当头、实字托底，不搞花拳绣腿，不做表面文章，争做“主攻手”、不当“二传手”，看准的事大胆干、定下的事抓到底，确保政府工作掷地有声、落地见效。

我们必须优化服务，提升效能。案无积卷、事不过夜。各级政府要深入推进“行政效能革命”，大力弘扬“马上就办、办就办好”的作风，优化政务工作流程，逐步推行“不见面审批”，下大气力精简会议文件，加强督查问责，着力提高办事效率和服务水平。我们必须廉洁从政，从严治政。打铁必须自身硬。各级政府要以铁一般的纪律，锻造铁一般的队伍，严格遵守党章党纪党规，贯彻落实中央八项规定实施细则，驰而不息改作风、纠“四风”，杜绝形式主义、官僚主义新表现，严厉整治发生在群众身边的腐败问题，以实际行动赢得人民信任和支持。

各位代表，时代出卷，我们答卷，人民阅卷。我们要以习近平新时代中国特色社会主义思想为指引，更加紧密地团结在以习近平同志为核心的党中央周围，在省委、省政府和市委的坚强领导下，不忘初心、牢记使命，开拓进取、真抓实干，为加快建设创新美丽幸福商洛、决胜全面小康社会而努力奋斗！
